# Annual Dynamics and Functional Traits of Viral Communities in Tropical Intertidal Sands of Sanya Bay

**DOI:** 10.3390/v18050500

**Published:** 2026-04-25

**Authors:** Zijia Wang, Zongminghan Liu, Juntao Zeng, Jiwei Li, Jiahao Cheng, Xiaoxue Qi, Jingwen Li, Shijie Bai

**Affiliations:** 1 Institute of Deep-Sea Science and Engineering, Chinese Academy of Sciences, Sanya 572000, China; 2 College of Marine Sciences, University of Chinese Academy of Sciences, Beijing 100049, China

**Keywords:** tropical intertidal sediments, viral community structure, metagenomics

## Abstract

Viruses are key regulators of marine microbial communities, yet their temporal dynamics in tropical intertidal sediments remain poorly characterized. We conducted a year-long metagenomic survey of sandy intertidal sediments in Sanya Bay (60 monthly samples from five sites) to examine viral taxonomy, community structure, lytic proteins, and auxiliary metabolic genes (AMGs). Within the classifiable fraction, the assemblages were consistently dominated by *Assiduviridae*. However, NMDS analysis revealed a significant overall seasonal shift, with October–December samples separating from the rest of the year. Co-occurrence network analysis identified five co-occurrence modules with distinct temporal patterns, alongside a concurrent decline in module abundance and lytic proteins in October. Functional annotation showed that cysteine and methionine metabolism, primarily driven by DNA methyltransferases, was identified as a highly represented AMG category among the annotated functions, while other pathways displayed seasonal variability. Collectively, these findings suggest that although characterized by a classifiable fraction dominated by *Assiduviridae*, the highly complex tropical intertidal viral communities undergo substantial seasonal reorganization in structure and functional potential.

## 1. Introduction

Viruses represent the most numerous biological entities in the marine environment, exhibiting an abundance approximately ten times that of prokaryotic organisms [1,2,3]. As a significant component of the microbial realm, viruses are associated with microbial adaptation and ecosystem functionality, while serving as primary drivers of marine biodiversity [4,5,6]. Viruses turn over 10% to 20% of the living biomass daily, a process that drives the viral shunt and shapes global biogeochemical cycles in tropical and subtropical regions by transforming organic matter into dissolved nutrients [4,7,8,9,10,11,12,13]. Furthermore, through horizontal gene transfer and host adaptation, viruses serve as key drivers of biodiversity and genetic innovation [14,15]. Viral metagenomics has extensively broadened our understanding of marine viruses, facilitating a deeper characterization of the temporal organization and functional profiles of viral communities within tropical coastal ecosystems [16].

Intertidal zones, functioning as vital ecological ecotones at the terrestrial-marine interface, represent one of the most widely distributed coastal ecosystems globally [17]. These environments are characterized by extreme environmental heterogeneity, where tidal oscillations trigger frequent and rapid transitions between aerobic and anoxic/suboxic states across minute-to-hour intervals and centimeter-scale spatial gradients [18,19,20]. Such dynamic physicochemical conditions necessitate flexible adaptive strategies from microbial inhabitants, resulting in community structures that are inherently more variable than those in other natural habitats [21,22]. Within these complex benthic systems, viruses act are associated with microbial population dynamics and community turnovers [23,24]. Previous studies indicated that the virus-to-prokaryote ratio (VPR) often exhibits insignificant seasonal patterns [25]. This stability is further reflected in the persistent presence of auxiliary metabolic genes (AMGs), which may contribute to viral maintenance across annual cycles [18]. While several studies have explored viral dynamics in intertidal sediments, systematic investigations specifically targeting tropical regions remain scarce [18,20,26,27,28,29]. This study aims to address this gap by providing a high-resolution, year-long perspective on viral community dynamics within a tropical intertidal habitat.

Located at the southern extremity of Hainan Island, Sanya Bay serves as a quintessential tropical coastal ecosystem characterized by its complex land–sea interactions and diverse benthic habitats [30]. The region maintains a stable thermal regime with an average annual air temperature of 25.5 °C, while experiencing distinct seasonal shifts in precipitation, with a pronounced rainy season from May to September [31]. Covering an area of approximately 120 km^2^, the bay’s environmental dynamics are primarily driven by the East Asian Monsoon system, which induces seasonal variations in vertical water mixing and nutrient distribution through alternating northeast and southwest monsoons [31,32]. These climatic factors, combined with significant terrestrial runoff from the Sanya River, create a highly heterogeneous environment that sustains extensive mangroves, coral reefs, and sandy intertidal shores [33]. While seasonal variations in planktonic communities and nutrient concentrations within the bay have been documented, viral biodiversity in these intertidal habitats remains unexplored [31,34,35].

In the present study, we performed a metagenomic assessment of viral communities within the tropical intertidal sands of Sanya Bay. We hypothesized that these viral communities potentially exhibit temporal variations in their structure and functional potential over an annual cycle. By analyzing 60 samples collected monthly from five representative sites over a complete annual cycle, this study provides a comprehensive characterization of the intertidal viral communities. Our investigation specifically focuses on: (1) defining the core taxonomic structure and the temporal persistence of dominant viral lineages; (2) elucidating the modular organization and key taxa within co-occurrence networks; (3) analyzing the temporal dynamics of the viral lytic machinery using the Prokaryotic DNA Virus Lytic Protein Dataset; and (4) resolving the AMGs and their potential involvement in mediating metabolic pathways. Collectively, this study provides a comprehensive perspective on the annual dynamics of tropical intertidal viruses, demonstrating that dramatic seasonal shifts in viral community structures are coupled with adjustments in their lytic machinery and auxiliary metabolic repertoires.

## 2. Materials and Methods

### 2.1. Sample Collection

**Figure 1:**
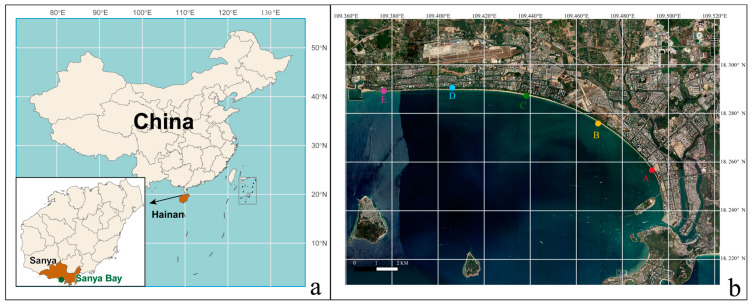
The Map of Sanya Bay and the sampling sites in this study. (**a**) Location of Sanya Bay, Hainan Province, China. (**b**) Layout of the Sanya Bay, showing the sampling sites (A, B, C, D, and E) along the coast line.

Surface intertidal sand samples were collected from five sites (A, B, C, D, and E) situated along the coast of Sanya Bay, Hainan Island, South China Sea. Sampling was conducted at monthly intervals from January to December in 2024, resulting in a total of 60 high-resolution temporal samples (Figure 1). At each sampling event, surface sediments (0–5 cm depth) were collected during low tide. The collected sand grains were immediately transferred into sample tubes placed in a portable cooler box with ice packs, and transported to the laboratory at a temperature of −20 °C. Upon arrival, samples were stored at −80 °C until analysis.

### 2.2. DNA Extraction and Sequencing

DNA was extracted from the samples using the DNeasy PowerSoil Pro Kit (QIAGEN, Germantown, MD, USA) in accordance with the provided instructions. DNA concentration was measured with a Qubit fluorometer (Invitrogen, Life Technologies Holdings Pte Ltd., Singapore). For metagenomic analysis, genomic DNA was fragmented to 350 bp fragments by sonication and ligated to Illumina adapters. Metagenomic libraries were then constructed with the NEBNext^®^ Ultra™ DNA Library Prep Kit for Illumina (NEB, Ipswich, MA, USA) following the standard protocol. Metagenomic sequencing was executed on the Illumina NovaSeq platform (PE150) at Novogene (Beijing, China), yielding approximately 20 Gb of raw paired-end data for each sample.

### 2.3. Metagenomic Processing and Viral Analyses

To process the raw metagenomic reads, adapter sequences were trimmed and low-quality data were filtered using fastp v0.23.4 [36]. Specifically, we discarded reads with an average quality score below 20 or a length shorter than 30 bp. The resulting high-quality reads were then subjected to de novo assembly via SPAdes v3.15.4 in meta mode. This assembly process included error correction and utilized a multi-k-mer strategy with values of 21, 33, and 55 [37]. Viral sequences were identified from assembled contigs with a minimum length of 1 kb using ViWrap v1.3.0 [38,39]. Viral prediction was primarily performed using VirSorter2 v2.2.4 and VIBRANT v1.2.1, and only contigs passing ViWrap default filtering criteria were retained for downstream analyses [40]. Viral scaffolds were binned into 943 viral metagenome-assembled genomes (vMAGs) using vRhyme v1.1.0 [41]. To reduce redundancy among viral genomes, vMAGs with a minimum length of 5 kb were dereplicated using dRep v3.6.2, based on a 95% ANI threshold, retaining 203 representative viral genomes for each cluster [42]. Viral taxonomy was inferred using PhaGCN v2.3.0 [43]. The vMAGs from each sample were mapped back to scaffolds using minimap2 v2.21, and coverage information was extracted using CoverM v0.7.0 [44,45]. For comparative analyses across samples, abundance values were normalized to account for differences in sequencing depth, and normalized viral abundance matrices were used for community composition, ordination, and network analyses. To characterize the functional potential of the viral community, AMGs were identified using DRAM v1.5.0 integrated within the ViWrap pipeline [46]. To minimize false positives and host contamination, a filtering which excluded genes located at scaffold ends, required viral-like flanking gene contexts, and removed genes belonging to COG categories T (Signal transduction) and B (Chromatin structure). Viral lytic proteins, including spanins, holins, and Endolysins, were annotated through HMMER v3.4 searches against the Prokaryotic DNA Virus Lytic Protein Dataset [47,48]. Downstream statistical analyses were conducted in R v4.5.2 [49]. Co-occurrence network was constructed based on Spearman correlations using ggClusterNet v2.0 [50]. vMAGs with a cumulative relative abundance of 1% across all 60 samples were retained. Only significant correlations (|r| > 0.6, BH-adjusted *p* < 0.05) were utilized to define network edges.

## 3. Results

**Figure 2:**
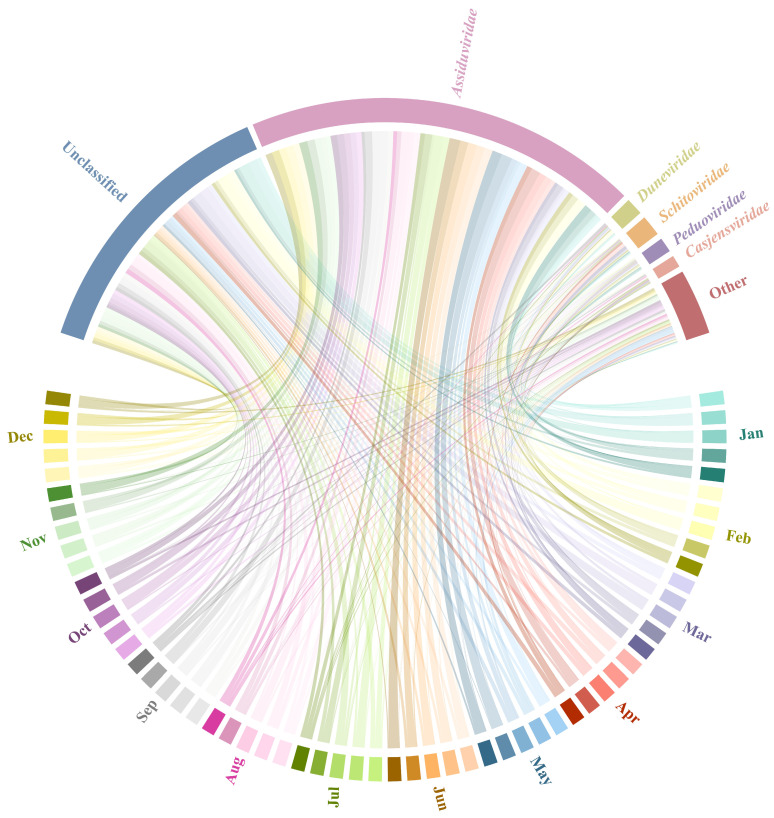
Family-level composition of viral communities across five sites in Sanya Bay over a 12-month period. Chord diagram showing the associations between samples and viral families. The ribbon widths indicate their relative abundances.

The viral community composition at the family level is presented in Figure 2. A substantial proportion of the recovered viral sequences could not be assigned to any known viral families. *Assiduviridae* was identified as the single most prevalent and abundant classified family, exhibiting a persistent presence across all sampling sites (A–E) and throughout the 12-month period. Its relative abundance remained high, suggesting that *Assiduviridae* appears to be a consistent feature within the classified viral community of the benthic viral seascape in Sanya Bay.

**Figure 3:**
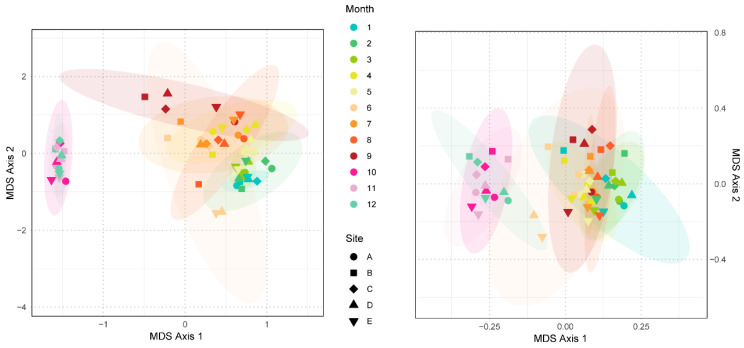
NMDS analysis of the virus community. The left panel uses Bray–Curtis dissimilarity (Stress = 0.24) and the right panel uses Jaccard distance (Stress = 0.19).

To further evaluate whether this taxonomic composition varied over space and time, we analyzed the beta-diversity using Non-metric Multidimensional Scaling (NMDS) based on both Bray–Curtis and Jaccard distances (Figure 3). A clustering was observed for the late samples, with October, November, and December grouping together and positioning separately from the other months. The remaining samples, representing January through September, formed a large assemblage. These patterns were statistically supported by PERMANOVA (R^2^ = 0.41, *p* = 0.001 for Month; R^2^ = 0.14, *p* = 0.001 for Site) and MRPP analysis confirming the separation (*p* = 0.004).

**Figure 4:**
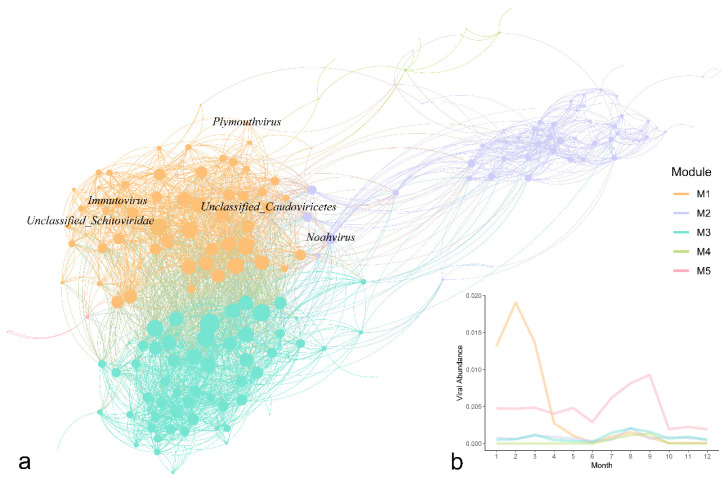
Network analysis and module distribution. (**a**) Co-occurrence network of viral community. Each node represents a vMAG, and edges indicate correlations between nodes. Nodes are colored by network module. The sizes of nodes reflect degree. (**b**) Monthly variations in the relative abundance of viral modules. Lines represent the average abundance of each module across samples collected over different months.

Building upon the temporal shifts identified in the NMDS analysis, the internal structure of these viral populations was explored. A co-occurrence network was constructed, which partitioned the community into five distinct co-occurrence modules (Figure 4a). In Module 1, *Plymouthvirus*, *Immutovirus*, *Unclassified_Caudoviricetes* and *Unclassified_Schitoviridae* were recognized as connectors (Zi ≤ 2.5, Pi > 0.62), while *Noahvirus* from Module 2 was recognized as module hub (Zi > 2.5, Pi ≤ 0.62) [51]. The relative abundance of these modules exhibited temporal fluctuations throughout the year (Figure 4b). Module 1 showed the most abundance in the early-year assemblage, reaching a sharp maximum during February before rapidly declining by April. Following the reduction in Module 1, Module 5 showed a steady increase from June, eventually peaking in September. A notable shift in the network state occurred in October, characterized by a decline in the abundance of all modules.

**Figure 5:**
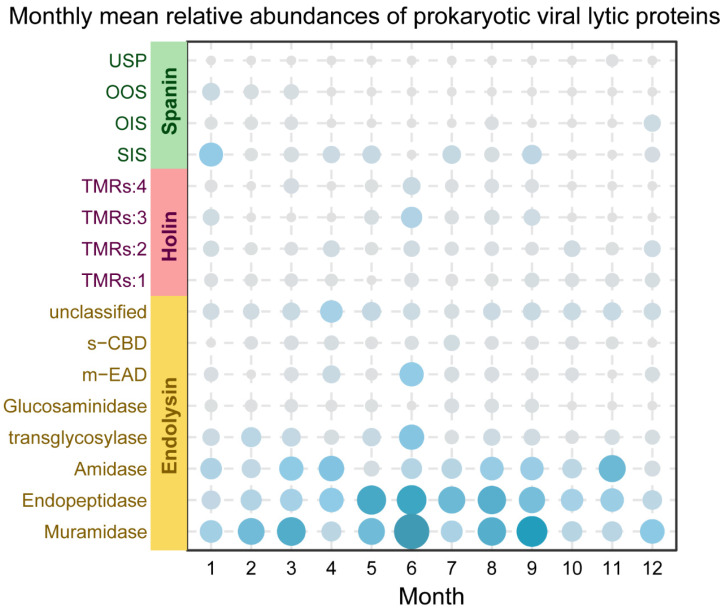
Monthly mean relative abundances of prokaryotic viral lytic proteins. The bubble size and color intensity represent the mean relative abundance of each protein category.

Given the shifts in community structure and network modules, the molecular machinery associated with host cell lysis was further examined. The abundance of viral proteins associated with host cell lysis was examined to assess the temporal dynamics of the lytic machinery (Figure 5). Overall, Endolysins were the most abundant category identified across all samples, with Muramidase and Endopeptidase maintaining the highest relative abundances throughout the 12-month period. While Endolysins displayed a clear seasonal trend, holins and spanins exhibited a more sporadic distribution without a well-defined monthly pattern. A general increase in lytic potential was observed from May to September. Specifically, June represented a peak period where multiple protein sub-categories, including transglycosylase, m-EAD, and Muramidase, reached their maximum annual values. A sharp reduction in the abundance of nearly all lytic protein categories was recorded in October, significantly lower than in September (Wilcoxon test, *p* = 0.016). During this period, the lytic potential dropped to its lowest recorded levels for most categories, aligning with the structural shifts observed in the community analyses.

**Figure 6:**
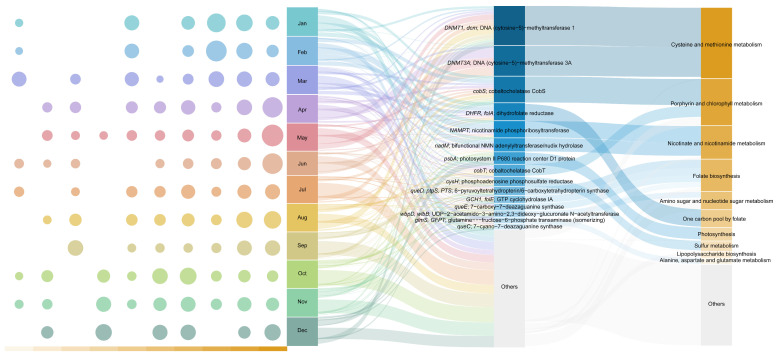
Seasonal dynamics and functional mapping of viral AMGs. The left bubble plot displays the relative abundance of the metabolic pathways across 12 months. Bubble size indicates the magnitude of relative abundance, while the color corresponds to the month. The bottom color strip identifies the pathways to match the Sankey diagram. The Sankey diagram illustrates the proportional flow from each month to the AMGs and their associated metabolic categories. The width of the flows represent relative abundance, normalized to the total number of predicted viral genes per sample. Only the top 15 AMGs and top 10 metabolic categories are individually labeled; remaining low-abundance features are aggregated as “Others”.

Finally, to understand how viruses manipulate host throughout these transitions, we characterized the viral AMGs and their associated metabolic pathways (Figure 6). Among the identified categories, cysteine and methionine metabolism was found to be the most frequently annotated pathway within our AMG dataset, primarily driven by DNA methyltransferase genes (*DNMT1* and *DNMT3A*). This pathway maintained a high and stable relative abundance throughout the year, although it reached an annual minimum in October, coinciding with the community structural shift observed in the NMDS analysis. The subsequent major functional categories included porphyrin and chlorophyll metabolism (dominated by *cobS* and *cobT*), which remained robust year-round, and nicotinate and nicotinamide metabolism (supported by *NAMPT* and *nadM*), the latter of which showed significant abundance between January and March. Additionally, pathways related to folate biosynthesis and the one-carbon pool by folate, associated with the *que* and *fol* gene families, displayed distinct temporal patterns: folate biosynthesis peaked in October, whereas the one-carbon pool remained relatively stable. Similarly, amino sugar metabolism, largely represented by the *wbp* gene family, also reached its maximum occupancy in October. Notably, certain pathways exhibited strong seasonal constraints. Photosynthesis (primarily *psbA*) was almost exclusively detected from October to December, while sulfur metabolism (primarily *cysH*) was restricted to the period between March and September. Among the top 10 pathways, lipopolysaccharide biosynthesis and alanine, aspartate, and glutamate metabolism exhibited the lowest relative abundances. The former showed a sharp increase during the final quarter of the year, while the latter remained consistently low. All functional groups outside the top 10 pathways and top 15 AMGs were aggregated into the “Others” category.

## 4. Discussion

Focusing on Sanya Bay as a representative tropical coastal ecosystem in China, this study provides a high-resolution, year-round metagenomic characterization of viral communities in intertidal sandy sediments, a habitat that remains poorly explored.

Annual monitoring in this study revealed that, within the classifiable fraction, *Assiduviridae* consistently dominated the known viral composition throughout the study area (Figure 2). Notably, while PERMANOVA identified significant spatial heterogeneity among sampling sites (R^2^ = 0.14, *p* = 0.001), this variation was secondary to the dominant monthly transitions (R^2^ = 0.41, *p* = 0.001).” According to the latest International Committee on Taxonomy of Viruses (ICTV) classification [52], this family belongs to Caudoviricetes and comprises three genera: *Nekkelsvirus*, *Cebadecemvirus*, and *Cellubavirus* [53]. Recent studies indicate widespread distribution of *Assiduviridae* across environments ranging from spring blooms in the North Sea [54] to deep-sea seamount sediments [55]. The primary hosts of *Assiduviridae* are Bacteroidetes, particularly the genus *Cellulophaga* [54,56]. These bacteria are key drivers in the degradation of algal polysaccharides and complex organic matter. In this typical tropical coastal environment, terrestrial runoff introduces abundant nutrients and maintains high primary productivity [34,57]. The continuous deposition of this biomass likely provides a rich source of organic matter for heterotrophic microorganisms in the sediments. The consistently high abundance of *Assiduviridae* throughout the year suggests a persistent potential for regulating organic-degrading bacterial populations, playing a crucial role in maintaining the steady state of sedimentary carbon cycling. Simultaneously, the coexistence of *Schitoviridae* and *Peduoviridae* across these sites reveals the viral community adaptation to microenvironmental heterogeneity. Recent research in membrane bioreactors has shown that these two families exhibit distinct redox-dependent dynamics: *Schitoviridae* tends to dominate under anaerobic conditions, while *Peduoviridae* favors aerobic environments [58]. Their coexistence is temporally aligned with the cyclical microenvironments of alternating aerobic and anaerobic conditions characteristic of the variable intertidal zone. Notably, a substantial number of viral sequences remain unclassified within this habitat. This finding aligns with recent discoveries in other intertidal habitats [59], indicating that vast amounts of unknown viral information persist even in common environments.

In the NMDS analysis of viral community structure (Figure 3), we found that the data can be broadly divided into two major clusters: one group covering January to September, and another group covering October to December. The first three quarters remained relatively stable, but October marked a distinct turning point, temporally coinciding with the monsoon transition (from the southwest to the northeast monsoon) and the typical alternation between wet and dry seasons [34,57,60]. The observed separation in community structure revealed by NMDS analysis is temporally aligned with this transition, suggesting that these seasonal shifts likely provide the ecological context for community reorganization. Concurrently, the co-occurrence network (Figure 4b) revealed a synchronous decline in all module abundances during October. It is important to note that correlation-based networks represent statistical associations whose structures can be driven by various factors, including actual ecological interactions, shared environmental filtering, or ecological niches [51,61,62]. Since such networks are primarily tools for generating hypotheses [63], we suggest that the simultaneous collapse of these modules does not necessarily indicate a breakdown of specific biotic interactions. Instead, it likely reflects a systemic community reorganization occurring amid the environmental changes associated with the monsoon transition. Similarly, topological hubs (e.g., *Noahvirus*) do not automatically constitute significant ecological importance. Their high connectivity may merely reflect their topological roles as ecological “generalists” [51,61]. Ultimately, these dramatic structural fluctuations in the viral community coincide with significant adjustments in their functional strategies.

In the viral metagenomic assemblages (MAGs) mined and identified from 60 intertidal sandy sediments in Sanya Bay, we analyzed the associated AMGs they harbored. Cysteine and methionine metabolism appeared to be predominant based on our functional profiling throughout the annual cycle, primarily driven by DNA methyltransferase genes (annotated as *DNMT1* and *DNMT3A*) (Figure 6). The widespread presence of these genes aligns with recent findings across diverse marine ecosystems [64,65,66]. For instance, DNA methyltransferases (DNMTs) have been identified as highly abundant AMGs in the Chinese continental shelf [64] and the Baltic Sea water column [65], while cysteine and methionine metabolism constituted the most prevalent functional category in the surface microlayer of the Central Arctic Ocean [66]. This widespread prevalence underscores a significant functional potential for viral-mediated DNA modification, which might in turn facilitate host adaptation and viral persistence across varying environmental conditions. Mechanistically, this pathway generates S-adenosylmethionine (SAM), an essential methyl donor for DNA methyltransferases [67]. The high prevalence of these genes may represent an adaptation to host restriction-modification (RM) systems. By methylating their own genomes, viruses can evade cleavage by host restriction endonucleases or maintain compatibility with host epigenetic patterns [68,69]. Additionally, the abundance of cysteine and methionine metabolism significantly decreased in October, consistent with shifts observed in NMDS analysis and network analysis. This trend likely reflects shifts in environmental parameters that alleviated the selective pressure for methylation-mediated viral defenses.

Notably, AMGs associated with folate metabolism were highly prevalent in our dataset, consistent with recent findings in diverse environments ranging from marine ecosystems to activated sludge treating antibiotic-production wastewater. This suggests the universal significance of folate-related pathways in the viral life cycle [70,71,72]. Folate and its derivatives, such as tetrahydrofolate (THF), serve as essential carriers of one-carbon units, driving a suite of critical biosynthetic reactions within host cells. These include the synthesis of purines, thymidine, and various amino acids (e.g., methionine, serine, and glycine), as well as the methylation of DNA, RNA, and proteins [73,74,75]. We revealed distinct seasonal dynamics between two folate-related pathways: AMGs involved in folate biosynthesis exhibited a significant increase in abundance from October to December, whereas the one-carbon pool by folate remained relatively stable throughout the year. The folate biosynthesis pathway in our study was primarily represented by genes such as *GCH1*/*fol*E, which initiates precursor synthesis, and *queC/D/E*, which are involved in tRNA hypermodification. Their primary function is to ensure a continuous supply of nucleotide precursors and optimize translation efficiency [73,74,76], thereby enabling viruses to maintain high replication rates even amidst host metabolic fluctuations. The enrichment of these genes may suggest an enhanced potential for viruses to modulate host folate-related pathways, potentially providing a mechanism to help hosts respond to environmental stress. Research indicates that folate possesses antioxidant properties, helping hosts mitigate oxidative damage induced by factors such as antibiotics [72,77]. Furthermore, an enrichment of folate synthesis genes in viral genomes has been observed under benzo[a]pyrene exposure [72,78]. In the bathypelagic zone, folate biosynthesis AMGs are significantly more abundant in oligotrophic free-living viral communities compared to those in nutrient-rich particle-attached (PA) environments; this is considered a key strategy for ensuring viral replication when host nutrients are limited [70]. Consequently, we hypothesize that the surge in folate biosynthesis AMGs from October to December represents an adaptive response to environmental shifts typically associated with the transition period that may exacerbate host metabolic stress. In contrast, the stable abundance of AMGs within the one-carbon pool by folate pathway (represented by genes such as DHFR) points to a consistent functional potential across the annual cycle. This pathway is putatively involved in the direct supply and cycling of one-carbon units, potentially serving as an important hub for nucleotide biosynthesis [73].

AMGs associated with amino sugar and nucleotide sugar metabolism also exhibited a pronounced abundance peak from October to December, with the *wbpD* gene being particularly prominent. The *wbpD* gene plays a crucial role in the biosynthesis of B-band O-antigen and lipopolysaccharide (LPS) precursors [79,80,81]. Interestingly, a synchronous enrichment of folate and amino sugar metabolism AMGs has also been observed in bathypelagic free-living viral communities [70]. This finding aligns with our observations during the monsoon transition in October, suggesting a potential for viral involvement in modulating host cell surface structures, which might represent a strategic response to environmental fluctuations. These genetic modifications could potentially contribute to enhanced host fitness and survivability under stress, thereby possibly offering a selective advantage for viral persistence and proliferation [82].

The photosynthesis pathway (primarily represented by *psbA*) exhibited strict seasonal variation, being detected almost exclusively between October and December. The viral *psbA* gene encodes the D1 protein, a core component of Photosystem II (PSII) that is highly susceptible to continuous photodamage. The high conservation of this gene in certain viral lineages suggests its critical role in maintaining host photosynthetic activity during the infection cycle [83,84]. Prior studies have confirmed that periods of high irradiance can indirectly promote the prevalence of *psbA*-carrying viruses [83,85,86]. In Sanya Bay, the enrichment of viral *psbA* during this period likely indicates a viral adaptation to fluctuating light conditions, thereby facilitating efficient viral proliferation. In stark contrast to the trends observed for photosynthesis genes, the sulfur metabolism pathway (primarily *cysH*) was detected only during the wet season (March to September). *cysH* is a key gene encoding the assimilatory sulfate reduction pathway and has been identified as a significant functional component in viruses inhabiting Chinese coastal intertidal flats [59,87]. More importantly, viral *cysH* is thought to streamline sulfur assimilation and assist the host in maintaining intracellular redox homeostasis. This functional repertoire suggests a potential for metabolic support, which might be advantageous for survival amidst the drastic hydrological and redox (Eh) fluctuations that are characteristic of intertidal sedimentary environments [88].

Viruses possess not only AMG that regulate host metabolism, but also another class of genes within their genomes that determine their ecological role. These genes encode proteins of the lytic system, which serve as indispensable components of the viral life cycle. By inducing host cell lysis, these proteins drive the flux of nutrients and genetic information throughout the ecosystem [48]. Viral lysis proteins are essential for releasing progeny viruses from host cells and typically comprise a three-component system: endolysins that degrade peptidoglycan cell walls, holins that form pores in the cytoplasmic membrane to allow endolysin passage, and spanins that disrupt the outer membrane [48]. Our analysis revealed (Figure 5) that endolysins, particularly those possessing muramidase and endopeptidase activities, constitute the most abundant category of lysis proteins throughout the year. This finding aligns with prevailing field consensus, where endolysins are recognized as indispensable core components of the phage lysis cycle [89,90]. The inconsistent patterns of holins and spanins compared to Endolysins likely stem from both biological and technical factors. Biologically, the shifting viral modules suggest that different lineages employ diverse or non-canonical lytic strategies. Technically, the high sequence divergence of holins and spanins makes them more difficult to detect than the more conserved catalytic domains of endolysins, even when using specialized databases. Thus, while total lytic potential remains a robust seasonal indicator, its internal composition reflects the inherent complexity and annotation constraints. The abundance of these lytic proteins exhibits a pronounced seasonal pattern, increasing significantly from May to September and peaking in June. This period coincides with the southwest monsoon (rainy season). We hypothesize that this enhanced lytic potential reflects the ‘Kill-the-Winner’ ecological model. In such nutrient-rich environments, fast-growing host populations reach high densities, creating optimal conditions for viruses to adopt an active lytic strategy [91,92,93]. However, the sharp decline in nearly all lytic protein categories in October primarily highlights a significant reduction in viral lytic potential. This trend, coinciding with the seasonal monsoon transition, suggests a potential adjustment in viral life strategies. Ultimately, these synchronized fluctuations in lytic and metabolic gene markers suggest that the viral community undergoes a substantial reorganization of its functional potential in October, consistent with the structural shifts identified in the NMDS analysis.

Beyond coastal ecology, the rapid wet-dry cycles and redox fluctuations of tropical intertidal zones make them valuable terrestrial analogues for studying potentially habitable ancient Martian lacustrine systems, such as those at Gale Crater, which experienced similar episodic drying [94]. Furthermore, active tidal interactions creating dynamic fluid-solid interfaces are predicted on ocean-bearing exoplanets [95,96]. In such highly variable habitats, the viral adaptive strategies observed in this study—specifically the shifts in lytic potential and the deployment of stress-responsive AMGs (e.g., DNA methyltransferases)—may serve as potential strategies for modulating host metabolism and survival [97]. Investigating these viral community dynamics and functional repertoires in extreme terrestrial analogs provides a crucial biological framework for understanding microbial resilience and identifying potential biosignatures in fluctuating extraterrestrial environments [94,98].

## 5. Conclusions

This study provides a year-round metagenomic characterization of viral communities in tropical intertidal sandy sediments of Sanya Bay. We observed a taxonomic composition dominated by *Assiduviridae* among the classifiable viral lineages, alongside clear seasonal shifts in community structure, network organization, and functional potential. Notably, October emerged as a transition period associated with reduced module abundance and decreased lytic protein representation. Functional analyses revealed that viral AMGs, especially those involved in cysteine and methionine metabolism, were consistently abundant, while several other pathways showed seasonal variability. Overall, our findings indicate that tropical intertidal viral communities undergo seasonally associated structural and functional adjustments, likely linked to monsoon-driven environmental changes. While specific known lineages such as *Assiduviridae* persist throughout the year, the substantial fraction of unclassified viruses suggests that the overall community complexity requires further exploration.

## Data Availability

Raw sequencing reads for all samples were deposited in the NCBI database (http://www.ncbi.nlm.nih.gov/, accessed on 22 March 2026) under BioProject accession number: PRJNA1431133 for the microbial metagenomic datasets of all the sediments in this study.
